# Multiplexed Data-Independent Acquisition-Based Proteomics Enabled by TMTpro Complementary Ions

**DOI:** 10.1021/acs.analchem.5c03563

**Published:** 2025-11-26

**Authors:** Zicong Wang, Peng-Kai Liu, Haiyan Lu, Lingjun Li

**Affiliations:** † School of Pharmacy, 5228 University of Wisconsin—Madison, Madison, Wisconsin 53705, United States; ‡ Biophysics Graduate Program, 5228 University of Wisconsin—Madison, Madison, Wisconsin 53705, United States; § Department of Chemistry, 5228 University of Wisconsin—Madison, Madison, Wisconsin 53706, United States; ∥ Lachman Institute for Pharmaceutical Development, School of Pharmacy, 5228 University of Wisconsin—Madison, Madison, Wisconsin 53705, United States; ⊥ Wisconsin Center for NanoBioSystems, School of Pharmacy, 5228 University of Wisconsin—Madison, Madison, Wisconsin 53705, United States

## Abstract

Data-independent acquisition (DIA) has emerged as a powerful approach in quantitative proteomics, offering more comprehensive and reproducible proteome coverage than the conventional data-dependent acquisition (DDA) method. However, applying multiplexed isobaric labeling to DIA has been challenging due to ratio distortion caused by coisolation and cofragmentation interference. Here, we present a 3-plex TMTpro complementary ion (TMTproC)-based DIA strategy that leverages complementary ions in isobaric labeling to achieve accurate quantification without increasing spectral complexity. By implementing a 4-Da spacing between complementary ions, we significantly reduce isotopic envelope overlap and simplify deconvolution. We systematically optimized higher-energy collisional dissociation (HCD) settings for complementary ion generation and validated this approach using tryptic bovine serum albumin (BSA) peptides labeled at 1:1:1, 10:5:1, and 1:5:10 ratios, achieving median peptide-level ratios within 10% of expected values and median coefficients of variation (CVs) below 4% across triplicates. We further demonstrated this method by applying TMTproC labeling across a 10-fold dynamic range to the yeast proteome in a strong human proteome background. The results exhibited high quantification precision and minimal ratio distortion. Overall, TMTproC-DIA provides a robust, versatile, and scalable solution for high-throughput DIA-based proteomics.

## Graphical abstract

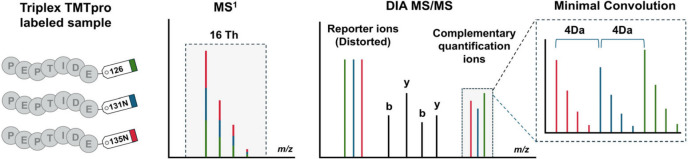

## Introduction

Quantitative proteomics plays an essential role in decoding complex biological systems and discovering disease biomarkers.^1–3^ Although data-dependent acquisition (DDA) remains popular for protein identification and quantification, its tendency to preferentially fragment high-abundance precursor ions can limit proteome coverage and result in high missing value rates between runs.^4,5^ To overcome these limitations, data-independent acquisition (DIA) has emerged as a transformative approach.^4,6,7^ DIA systematically fragments all precursors within predefined large *m*/*z* windows, generating unbiased MS/MS spectra.^8,9^ This systematic fragmentation enhances coverage and reproducibility, establishing DIA as an indispensable tool in modern proteomics.^7,10^

Label-free DIA quantification, however, can still suffer from variability introduced during sample preparation, injection, and chromatographic retention-time alignment.^11^ Multiplexed proteomics using stable isotope labeling - most prominently via isobaric labeling - ameliorates these sources of error by allowing multiple samples to be combined and analyzed in a single LC-MS run, thereby boosting throughput and reproducibility while reducing the required instrument time.^11–16^ Although widely used in DDA workflows, isobaric tags such as Tandem Mass Tags (TMT)^17–19^ and *N*, *N*-Dimethyl-Leucine (DiLeu)^20–25^ are susceptible to ratio distortion. When coisolated peptides are present, the reporter ions experience interference from cofragmented precursors.^26,27^ Under DIA conditions, this interference becomes more pronounced due to the large isolation windows, which increase the likelihood of coisolation and cofragmentation.^28–30^ These factors compromise the accuracy of isobaric labeling and consequently limit its applicability in DIA workflows.

Several alternatives have been explored to achieve multiplexing with DIA. Nonisobaric labeling strategies, exemplified by the plexDIA approach, circumvent some of these challenges by incorporating distinct isotopic mass increments (e.g., mTRAQ tags) for each sample.^31–34^ However, nonisobaric labeling considerably increases spectral complexity at both the MS1 and MS2 levels. The resulting overlapping peptide fragments further complicate data analysis and demand sophisticated computational tools for peptide identification.^34^ This complexity escalates with the number of multiplexed samples, constraining the scalability of this method.^11,28^ Mass defect-based strategies offer another solution by introducing mDa level mass differences to reduce spectral complexity,^28,35–37^ but they require ultrahigh-resolution instrumentation and extended cycle times, thus limiting proteome coverage.

Complementary-ion-based quantification offers a promising solution to overcome these limitations. During high-energy collisional dissociation (HCD) of an isobaric-tagged peptide, the loss of the reporter group and neutral CO yields high-*m*/*z* complementary ions that retain the whole peptide backbone and channel-specific mass information.^38–41^ Unlike low-mass reporter ions that are identical across peptides, complementary ions are peptide-specific and remain distinguishable even when coisolation occurs, making them inherently suitable for DIA-based workflows.^30^

Prior studies, such as the Ac-AG tag-based method by Tian et al.,^30^ have demonstrated the feasibility of complementary-ion quantification in multiplex DIA experiments. However, this method faces limitations: the 1 Da spacing between complementary ions leads to extensive peak overlap in their isotopic envelopes.^30^ This overlap necessitates complex deconvolution processes to retrieve quantitative ratios, which has been reported to reduce the precision of quantification.^39^ Additionally, complete isotopic envelopes are required for effective deconvolution, further restricting proteome coverage. Ultranarrow (<0.5 Th) precursor isolation windows can alleviate overlap by selecting monoisotopic precursor peak in DDA mode,^39–41^ yet are impractical for DIA analysis.

Here, we present a novel 3-plex TMTpro complementary-ion (TMTproC)-based DIA strategy that directly addresses these issues. By combining the multiplexing capacity of isobaric labeling with the peptide specificity of complementary ions, this approach achieves accurate and precise quantification without increasing spectral complexity. To enhance quantification accuracy, complementary ions were designed with 4 Da spacing, effectively minimizing isotopic peak overlaps and simplifying deconvolution processes. We employed MSFragger-DIA for library-free DIA searches, streamlining the workflow by eliminating the need for building spectral libraries.^42^ Using triply labeled bovine serum albumin (BSA) standards mixed at defined ratios and a human-yeast dual-proteome model, we demonstrate that TMTproC-DIA delivers accurate, precise, and interference-resistant quantification while maintaining broad proteome coverage, positioning it as a practical, high-throughput solution for multiplexed DIA proteomics.

## Materials and Methods

### Chemicals and Materials

LC/MS-grade acetonitrile (ACN) and water (H_2_O), formic acid (FA), trifluoroacetic acid (TFA), Pierce HeLa Protein Digest Standard, Pierce BSA Protein Digest (MS grade), 1 M triethylammonium bicarbonate (TEAB) buffer, 50% hydroxylamine solution, EasyPep peptide cleanup spin columns, and TMTpro 18-plex label reagents were purchased from Thermo Fisher Scientific (Pittsburgh, PA). Standard peptides were synthesized by GenScript Biotech (Piscataway, NJ). Mass spectrometry-compatible yeast digests were obtained from Promega (Madison, WI). Fused silica capillary tubes (inner diameter 75 μm, outer diameter 375 μm) were purchased from Polymicro Technologies (Phoenix, AZ).

### TMTpro Labeling

TMTpro labels 126, 131N, and 135N were dissolved in anhydrous acetonitrile (ACN) at a concentration of 4 μg/μL. Digested peptide samples were dissolved in 100 mM triethylammonium bicarbonate (TEAB) buffer and mixed with TMTpro at a peptide-to-label ratio of 1:10. The mixture was incubated at room temperature for 1 hour. Then, 5% hydroxylamine was added to quench the reaction, followed by incubation at room temperature for 15 minutes. Subsequently, samples labeled with different channels were combined. The combined samples were dried *in vacuo* using a SpeedVac, redissolved in 150 μL of 5% TFA, and desalted using EasyPep peptide cleanup spin columns according to the manufacturer’s protocol. The eluates were dried again using SpeedVac and then redissolved in H_2_O with 0.1% FA for LC-MS/MS analysis.

### LC-MS Analysis

Samples were analyzed using a Vanquish Neo UHPLC system (Thermo Fisher Scientific) coupled to an Orbitrap Exploris 480 mass spectrometer (Thermo Fisher Scientific). Peptides were loaded onto an in-house packed capillary column (75 μm × 25 cm) packed with 1.7 μm, 130 Å BEH C18 material (Waters). The mobile phase flow rate was set at 300 nL/min, with buffer A consisting of 0.1% FA in water and buffer B of 0.1% FA in 80% ACN. Peptide separation was performed using a 120 min gradient from 7% B to 30% B.

The mass spectrometry parameters were as follows: Data were acquired in positive ion mode. Full MS survey scans were acquired in the *m*/*z* range of 450–1,350 with an Orbitrap resolution of 60,000 at *m*/*z* 200. The normalized automatic gain control (AGC) target was set to 300%, with a maximum injection time of 100 milliseconds. The full MS scan was followed by 60 DIA scans with an isolation window of 16 Th (1 Th overlap between windows). The isolation windows were generated using the method editor in Xcalibur software, set at 15 *m*/*z* wide from *m*/*z* 450 to 1,350 with a 1 *m*/*z* overlap. MS/MS DIA scans were acquired over the *m*/*z* range of 120–2,550. A normalized AGC target of 2,000%, a resolution of 60,000 at *m*/*z* 200, and a maximum injection time of 120 milliseconds were used. Fragmentation was performed using HCD with a normalized collision energy (NCE) of 29%.

### Data Analysis

The data processing workflow was illustrated in Figure S1. Data analysis was performed using MSFragger (version 4.1),^43,44^ Philosopher (version 5.1),^45^ and FragPipe (version 22.0)^43,44^ through MSFragger-DIA^42^ library-free DIA searches. The reviewed *Homo sapiens* proteome database (downloaded on November 2, 2024; UniProt UP000005640; 41,312 entries including 20,656 decoys) was used for HeLa cell proteomics searches. For human-yeast two-proteome searches, a combined database of *Homo sapiens* and *Saccharomyces cerevisiae* (downloaded on November 2, 2024; UniProt UP000002311) was utilized, totaling 53,432 entries including 26,716 decoys. Precursor and fragment mass tolerances were set at ± 20 ppm. Variable modifications included methionine oxidation and protein N-terminal acetylation (up to five modifications per peptide, maximum of five combinations). Fixed modifications were set for cysteine carbamidomethylation and either TMTpro zero (+295.1896 Da) or TMTpro (+304.2072 Da) labeling on lysine residues and peptide N-termini. The DIA results were filtered to a 1% false discovery rate (FDR) at both the peptide and protein levels.

The search results from FragPipe were further analyzed for complementary ions using an in-house developed Python script. The Python script is available on Zenodo (10.5281/zenodo.14567079). Specifically, raw mass spectrometry data files (.raw) were converted to Python-readable .mzML files using MSConvertGUI (ProteoWizard).^46^ The exported PSM.tsv file from FragPipe contains peptide-spectrum matches (PSMs) and protein assignments. The theoretical *m*/*z* was calculated for each complement ion according to matched peptide mass (as shown in Supplementary Methods), with a mass error tolerance of 20 ppm applied during matching. Using scan numbers, the PSMs were associated with the corresponding peptide-coupled complementary ion intensities. The matched intensities were then used for peptide and protein quantification. In the 3-plex experiment, only PSMs where all three complementary ions detected were kept for quantification.

The obtained complementary ion intensity values were deconvoluted for [M+4] peak interference to eliminate intensity contributions of the [M+4] peak from the preceding complementary ion. The correction ratio was calculated from the molecular formula of the identified peptide. Then, isotopic tag purity correction was also performed according to the purity of the TMTpro isobaric tags (as detailed in Supplementary Methods). After correction, two filters were applied: (1) an intensity cutoff filter of 5*10^3^ was applied for all complementary ions to enhance quantification reliability. (2) Each complementary ion was required to exhibit [M+1] isotopic pattern to reduce misidentification from background noise. [M+1] isotopic ions were identified with a mass error tolerance of 20 ppm and an intensity cutoff filter of 5*10^2^. After filtering, peptide ratios were calculated from the three PSMs with the highest total complementary ion intensities, and protein ratios were calculated by a hyperscore - weighted average of the three unique peptides with the highest total complementary ion intensities. The measured quantification ratios were calculated by normalizing the intensity of each isobaric channel with weighted average intensity of all three channels, as described in previous reports.^29,30^ The MS data have been deposited in the ProteomeXchange consortium via the MassIVE repository with the accession code PXD069097.

MS spectrum was analyzed and extracted using Xcalibur 4.0 software. Graphs and statistical analysis were made with GraphPad Prism 10.3.1 and Origin 2024b.

## Results

### TMTproC-DIA Enables Accurate, Interference-Resistant Multiplexed DIA Analysis

We developed the TMTpro complementary-ion (TMTproC)-DIA strategy to enable multiplexed DIA analysis that achieves accurate, interference-resistant quantification without increasing spectral complexity. This approach leverages the distinct properties of complementary ions generated during MS/MS fragmentation to overcome the limitations of conventional reporter-ion-based quantification. Unlike low-mass reporter ions, which are identical across peptides and prone to coisolation interference, complementary ions encode both peptide-specific and label-specific information. This unique property ensures accurate quantification even in the presence of coisolated peptides, making it suitable for DIA-based applications (Figure 1).

**Figure 1:**
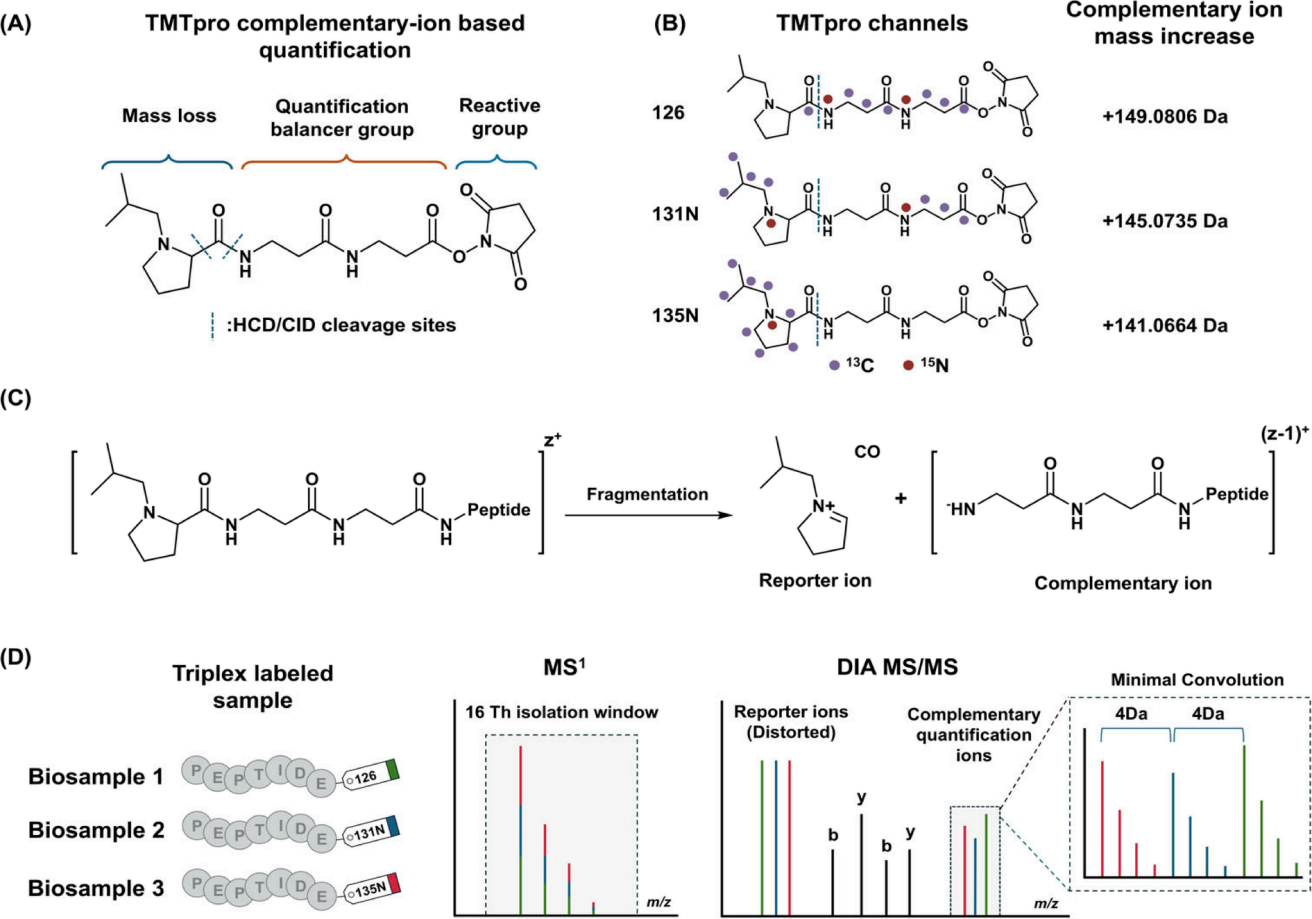
Schematic overview of the TMTpro complementary ion-DIA-based multiplexed proteomics. (A) Structure of the TMTpro reagent, highlighting functional regions critical for complementary-ion-based quantification: the mass loss region (reporter ion + neutral loss CO), quantification balancer group (two β-alanine residues that are linked to peptide in the complementary ion), and the amine-reactive NHS ester group. HCD/CID fragmentation sites are indicated in the dashed line. (B) Isotopic composition of the three TMTpro channels (126, 131N, and 135N) used in this study, which introduces distinct mass increase of +149.0806, +145.0735, and +141.0664 Da to peptide when forming complementary ions. (C) Formation of complementary ions upon HCD fragmentation. The peptide-bound TMTpro tag loses a reporter ion and a neutral CO molecule, leaving a complementary ion that retains peptide-specific isotopic information for accurate quantification. (D) Schematic of triplex TMTproC-DIA analysis. In DIA mode, wide isolation windows (e.g., 16 Th) coisolate multiple precursors. Reporter ions at low *m*/*z* are easily distorted by coisolation interference, whereas complementary ions spaced by 4 Da minimize isotopic overlap, simplifying deconvolution and enabling interference-resistant quantification.

Central to this strategy is the TMTpro reagent (Figure 1A), a commercially available isobaric tag that reliably generates complementary ions upon HCD fragmentation.^19,40^ During fragmentation, the tag cleaves at specific bond sites, resulting in the loss of a reporter ion and a neutral CO molecule, leaving behind a complementary ion that retains the whole peptide backbone conjugated to the isotopically labeled two beta-alanine balancer group (Figure 1C).^40^

For our experiments, we selected three TMTpro channels (126, 131N, and 135N), each imparting distinct mass increments (+149.0806 Da, +145.0735 Da, and +141.0664 Da, respectively) to the peptide during complementary ion formation (Figure 1B). This carefully chosen set of channels produces a 4 Da spacing among complementary ions, minimizing isotopic overlap and reducing the need for complex deconvolution (Figure 1D). This advantage is further demonstrated in Figure S2, where theoretical complementary ion isotopic envelope convolution at different labeling ratios (1:1:1, 1:5:10, and 10:5:1) is compared between complementary ions with 1 and 4 Da spacing. While 1 Da spacing introduces substantial overlap requiring complex deconvolution, the 4 Da spacing effectively mitigates this issue and only requires the removal of slight [M+4] peak interference.

To validate this approach, we analyzed two tryptic peptide standards, DVGVLK and ANLSIK, with precursor *m*/*z* values of 619.9021 and 627.4113, respectively. These peptides, both carrying a + 2 charge, were designed to fall within the same DIA isolation window (16 Th centered at *m*/*z* 623.65, as shown in Figure 2A). DVGVLK was labeled at a 135N:131N:126 ratio of 1:5:10, while ANLSIK was labeled at the reverse ratio of 10:5:1. The peptides were mixed and analyzed to simulate the challenges of coisolation and cofragmentation encountered in DIA workflows.

**Figure 2:**
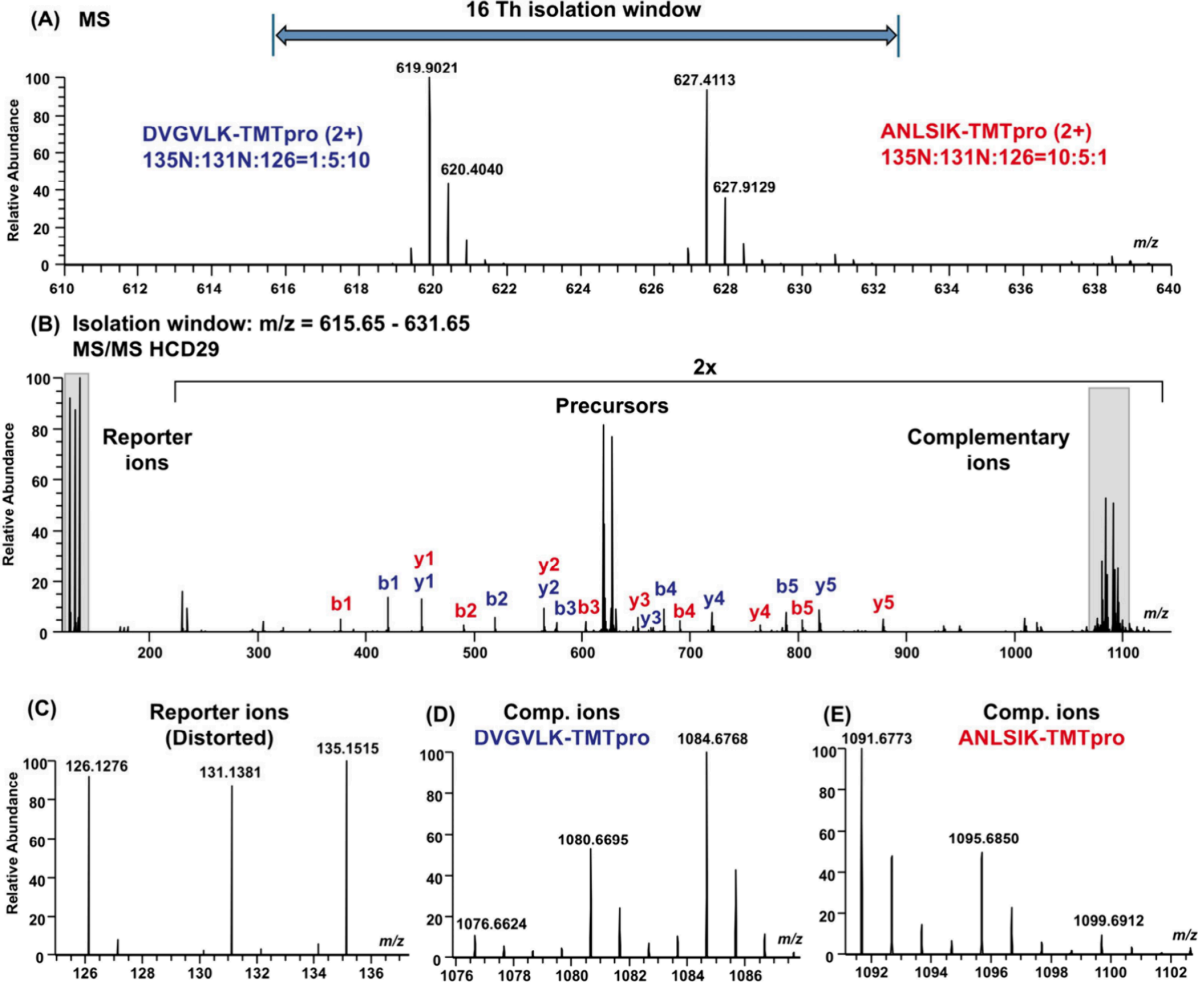
Validation of TMTpro complementary ion-DIA method using standard peptides. (A) MS1 spectrum showing coisolation of DVGVLK-TMTpro (labeled at 10:5:1) and ANLSIK-TMTpro (labeled at 1:5:10) percusor ions within a 16 Th isolation window centered at *m*/*z* 623.65. (B) Representative MS/MS spectrum acquired at an NCE of 29%. The spectrum illustrates reporter ions (low *m*/*z*, highlighted in the shaded region), peptide b/y fragment ions, and complementary ions (high *m*/*z* highlighted in the shaded region). (C) Detailed view of the reporter ion region. Quantification using reporter ions demonstrates significant ratio distortion due to coisolation interference, leading to near 1:1:1 ratio. (D, E) Detailed view of complementary ion region. Complementary ions for DVGVLK-TMTpro (D) and ANLSIK-TMTpro (E) show accurate representation of theoretical labeling ratios (1:5:10 and 10:5:1).

As illustrated in Figure 2B, both peptides were coisolated and cofragmented, producing abundant b/y fragment ions in a single MS/MS spectrum. Conventional low-mass reporter ions displayed severe ratio distortion, yielding near 1:1:1 ratios (Figure 2C) due to coisolation interference. In contrast, the complementary ions, observed at higher *m*/*z*, accurately reproduced the intended labeling ratios (Figure 2D and E). This result confirms that the TMTproC-DIA strategy effectively mitigates interference, providing reliable, multiplexed DIA quantification.

### Establishing the Search Workflow for TMTpro Complementary Ions

To implement the TMTproC-DIA strategy, we established a customized workflow optimized for complementary ion analysis. First, MS raw data files were processed using MSFragger-DIA for library-free DIA searches,^42^ which eliminates the need to build spectral libraries and expands peptide identification beyond library constraints.^30^ Because TMTpro labeling yields identical backbone fragments across channels, the triplex data can be directly applied to any conventional DIA search pipeline without modification. Next, the resulting PSMs were analyzed using a custom Python script, which extracted triplex complementary ion intensities within a 20 ppm mass tolerance. Although the 4-Da spacing between complementary ions minimizes isotopic overlap, interference from the [M+4] isotopic peak can still occur - especially for higher-mass precursors or those with large intensity differences. To mitigate this, we calculated each peptide’s theoretical [M+4] intensity based on its molecular formula and subtracted it from the affected complementary ions, thereby ensuring more accurate quantification.

To further improve the reliability of complementary ion quantification, we applied additional data filtering criteria. In DIA, a 1-Da overlap between adjacent isolation windows ensures that the [M+1] isotopic peak is consistently coisolated and cofragmented alongside the [M+0] peak. As a result, both [M+0] and [M+1] isotopic peaks reliably appear in the complementary ion cluster (Figure S3). We therefore required the presence of the [M+1] isotopic peak to confirm that detected signals truly represent complementary ions rather than background noise. This filtering step helped ensure the inclusion of true complementary ions in the final data set.

### Optimizing HCD Fragmentation Energy for Complementary Ion Formation

We next optimized the higher-energy collisional dissociation (HCD) fragmentation energy to maximize the efficiency of complementary ion generation. Using peptide standards DVGVLK and ANLSIK, we evaluated complementary ion intensities across normalized collision energy (NCE) settings ranging from 25% to 35% in 2-unit increments. We measured the ratio of complementary ion intensity to precursor ion intensity to determine the optimal conditions. Complementary ion generation peaked at an NCE of 29% (Figure S4), consistent with previously reported optimal energy levels.^40^

To validate the selected NCE across a more diverse peptide set, we labeled tryptic HeLa peptide digests with TMTpro Zero and evaluated complementary ion generation at NCE values of 27%, 29%, 31%, and 33%. In Figure 3A, we show the complementary ion generation ratio at the PSM level, defined as the proportion of PSMs with quantifiable complementary ions relative to the total number of identified PSMs. The results showed a peak ratio of 44% at NCE 29%, representing the highest proportion of complementary-ion-containing PSMs. Protein-level statistics mirrored this trend: NCE 29% yielded 3,134 protein identifications, of which 2,645 were quantifiable via complementary ions (Figure 3B). This result represents the highest number of protein quantifications among the tested conditions. The distribution of the number of quantifiable peptides per protein across different HCD collision energies is shown in Figure S5. Together, these findings confirm that NCE 29% optimizes complementary ion generation at both the PSM and protein levels, which is modestly lower than the NCE 35% recommended for TMTpro reporter-ion quantification on the same instrument.^47^ Consequently, we applied NCE 29% in all subsequent experiments.

**Figure 3:**
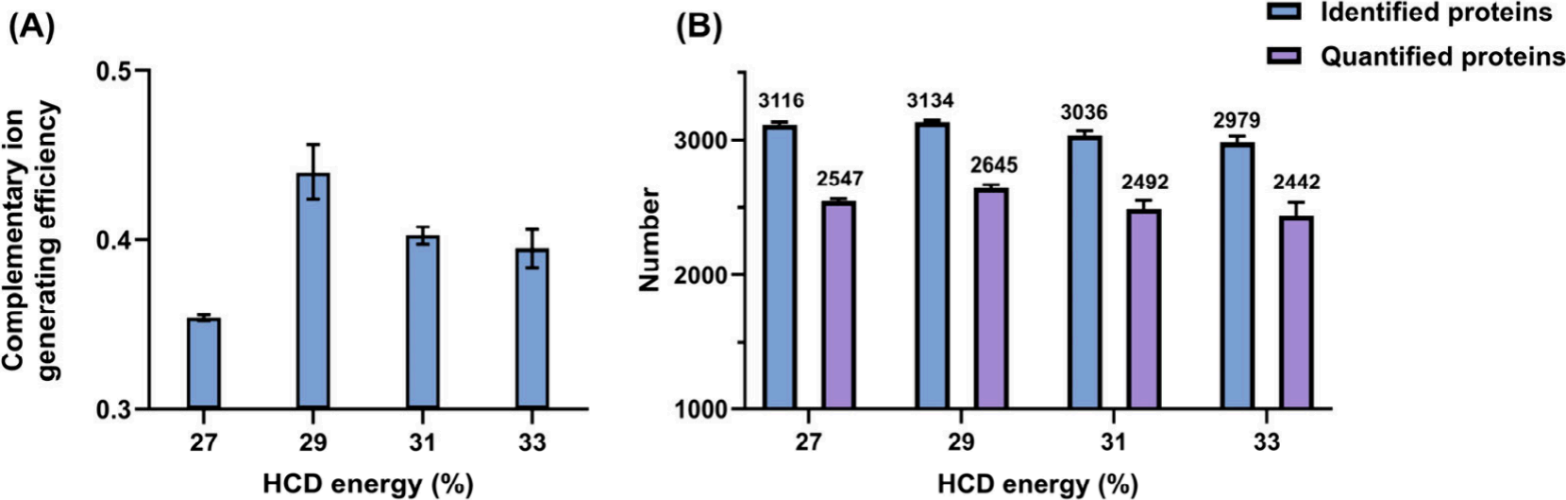
Optimization of HCD energy for complementary ion generation. (A) Efficiency of complementary ion generation at the PSM level for different HCD energy settings (NCE 27%, 29%, 31%, and 33%). Efficiency is defined as the proportion of PSMs with quantifiable complementary ions among total identified PSMs. The highest ratio (44%) was obtained at NCE 29%. (B) Protein-level results under different NCE conditions. The bar graph indicates the total number of identified proteins (blue) and quantified proteins using complementary ions (purple). Error bars represent the standard deviation across technical replicates.

### TMTproC-DIA Analysis of Triplex-Labeled BSA Sample

Building on the optimized HCD conditions, we applied the TMTproC-DIA workflow to evaluate its quantitative accuracy and precision. Tryptic BSA peptides were labeled with triplex TMTpro tags and mixed at ratios of 1:1:1, 10:5:1, and 1:5:10 (126:131N:135N). These samples were analyzed in DIA mode using LC-MS/MS to compare theoretical and measured ratios, thereby assessing quantification accuracy.

For the 1:1:1 sample, the median measured peptide-level ratio was 0.98:1.02:1.00 (Figure 4A), indicating excellent agreement with theoretical values. Across triplicates, the median coefficient of variation (CV) was 3.56%, as illustrated in the probability density plot (Figure 4B). This low median CV is comparable to that reported for the narrow isolation window-based TMTc method (6%) and significantly better than the full envelope-isolation-based TMTc method (16%).^39^ For the 10:5:1 and 1:5:10 samples, the median peptide-level ratios were 9.89:5.06:1.03 and 1.07:5.09:9.81, respectively (Figure 4C and D). Across triplicates, the median CVs for both samples were also below 4%. Thus, the 4-Da spacing between complementary ions proved essential for minimizing isotopic overlap and enhancing quantification robustness. A representative MS/MS spectrum of the peptide LGEYGFQNALIVR at various labeling ratios is shown in Figure S6. The spectrum demonstrates a high level of consistency between the measured relative intensities of complementary ions and their theoretical ratios.

**Figure 4:**
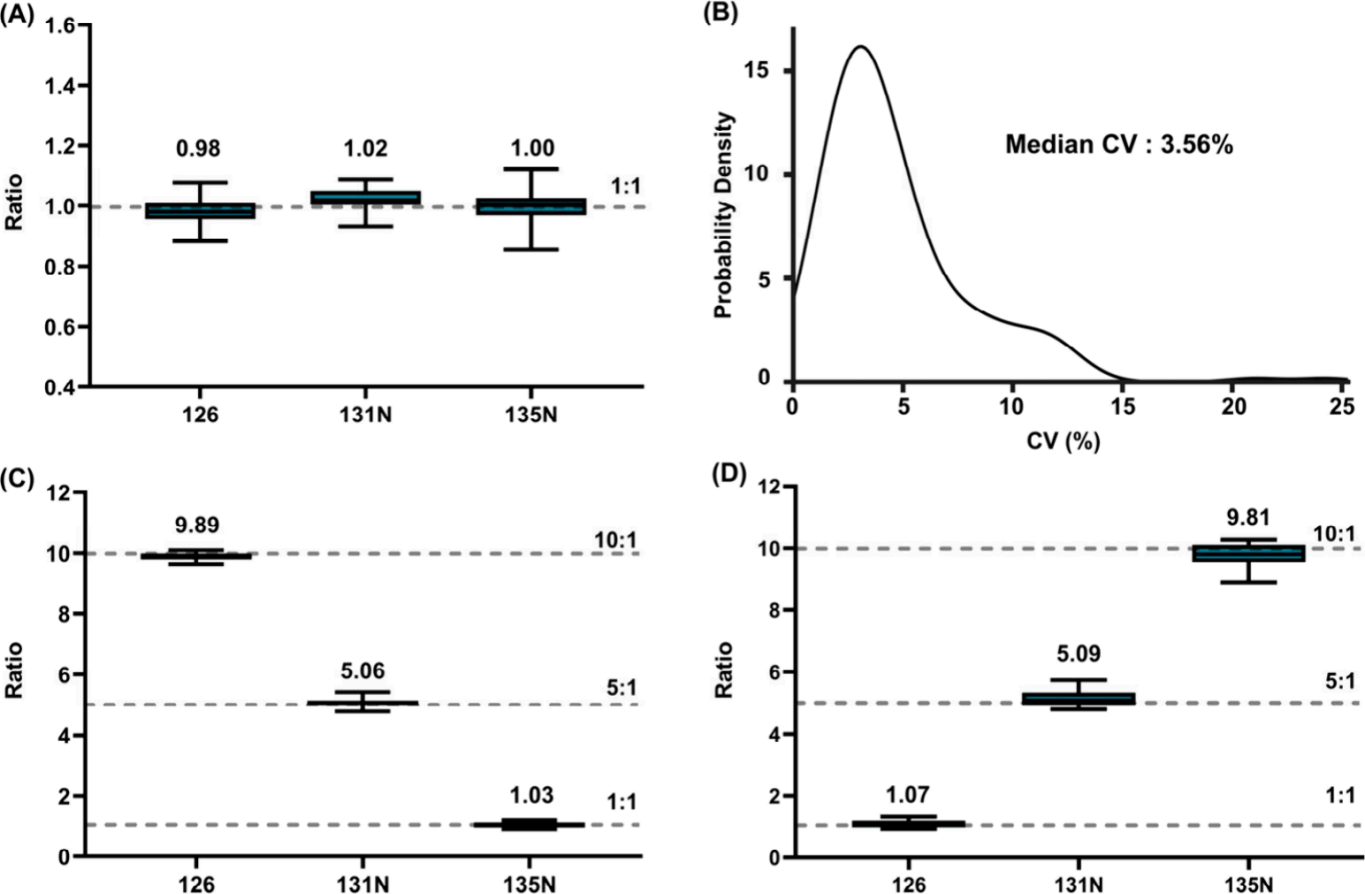
Quantitative accuracy and precision of TMTproC-DIA in triplex-labeled BSA peptides. (A) Box plot of peptide-level quantified ratios for a 1:1:1 labeled BSA sample across TMTpro channels (126, 131N, 135N). The dashed line indicates theoretical ratios. (B) Probability density distribution of CV values for the 1:1:1 sample across triplicates. The median CV was 3.56%, indicating high precision. (C, D) Box plots of peptide-level quantified ratios for BSA samples labeled at 10:5:1 (C) and 1:5:10 (D) across TMTpro channels. The dashed line indicates theoretical ratios. Box plots demarcate the median (line), the 25th and 75th percentile (box), and the fifth and 95th percentile (whiskers).

Notably, previous complementary ion quantification workflows often excluded peptides with charge states higher than 3+ due to challenges in isolating [M+0] peaks and subsequent deconvolution.^39–41^ In contrast, our workflow is fully compatible with high-charge-state peptides. As demonstrated in Figure S7, the 4+ charged peptide QEPERNE­CFLSHK from BSA was accurately quantified. This improvement enhances coverage and increases the depth of proteomic analysis.

These results confirm the high quantitative accuracy of TMTproC-DIA, with relative errors below 10% across a dynamic range spanning an order of magnitude. The method also exhibits outstanding precision, maintaining CVs below 4% across triplicates, even for complex labeling ratios. These findings highlight the reliability of the TMTproC-DIA workflow for multiplexed quantitative proteomics.

### TMTproC-DIA Analysis of a Two-Proteome Model

To further evaluate the quantitative accuracy and robustness of the TMTproC-DIA strategy, we applied it to a two-proteome model composed of HeLa cell (human) and *Saccharomyces cerevisiae* (yeast) lysates. HeLa peptides were labeled with TMTpro tags at a 1:1:1 ratio, while yeast peptides were labeled at a 1:5:10 ratio (126:131N:135N). The labeled lysates were combined at a yeast: HeLa ratio of 1:3 to simulate the quantification of low-abundance yeast peptides in a high-abundance HeLa background. The samples were then analyzed using nanoLC-DIA MS/MS on an Orbitrap Exploris 480 instrument (Figure 5A).

**Figure 5:**
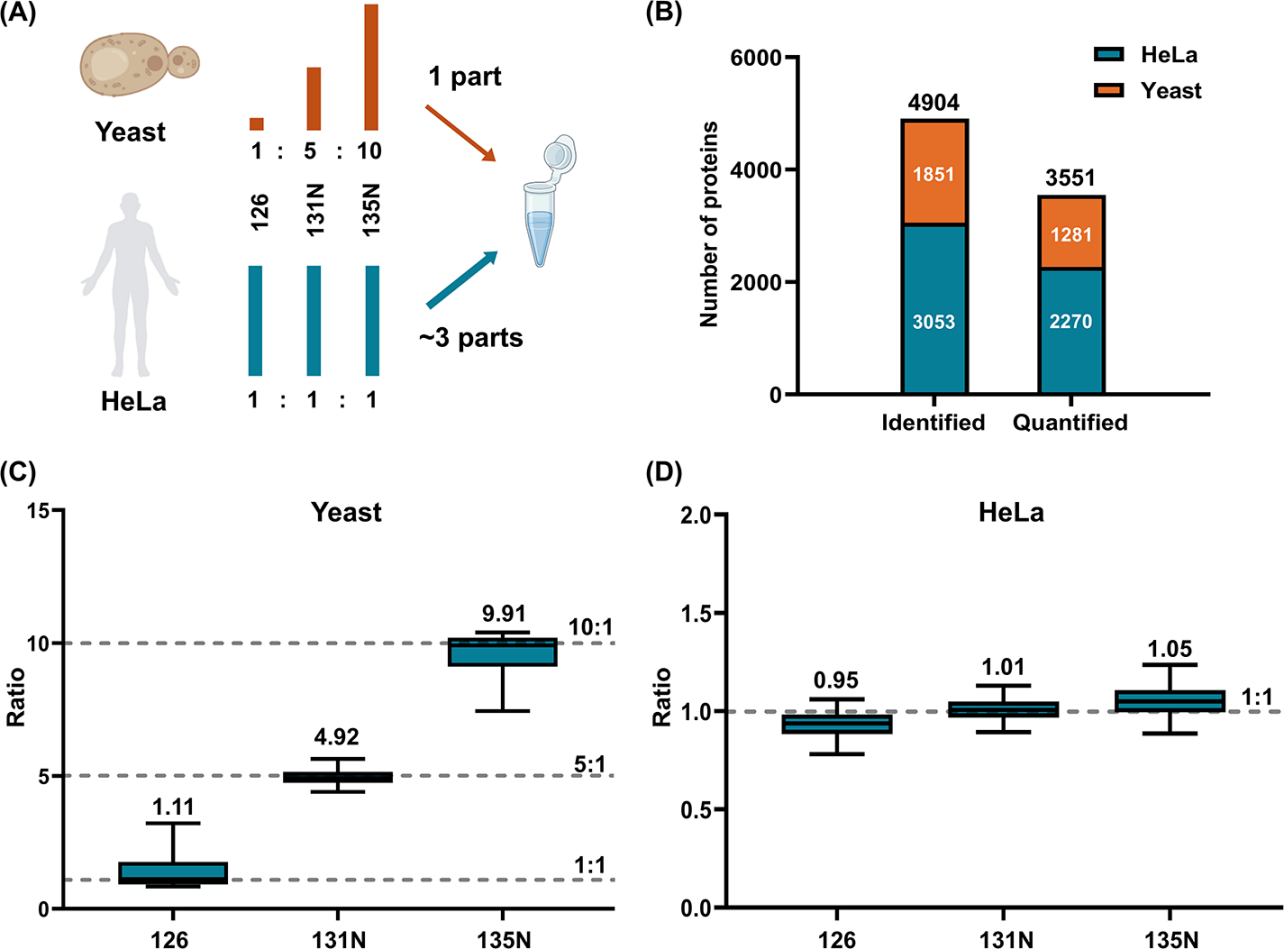
Quantitative accuracy and precision of TMTproC-DIA in human-yeast dual-proteome sample. (A) Schematic of the dual-proteome experimental design. Yeast peptides were labeled with TMTpro tags at a 1:5:10 ratio (126:131N:135N), while HeLa peptides were labeled at a 1:1:1 ratio. The labeled peptides were mixed at a yeast-to-HeLa ratio of 1:3 and analyzed using DIA mode. The icons were created with Biorender.com. (B) Bar chart showing the total number of identified and quantified proteins. (C) Box plots of quantified ratios for yeast proteins. (D) Box plots of quantified ratios for HeLa proteins. The dashed line indicates theoretical ratios.

In total, we identified 4,904 proteins, of which 3,551 were reliably quantified using complementary ions (Figure 5B). The distribution of quantified PSMs across *m*/*z* confirmed that the DIA mass range settings were appropriate (Figure S8). For yeast proteins, the median quantified ratios were 1.11:4.92:9.91, while for HeLa proteins they were 0.95:1.01:1.05, both closely reflecting their respective theoretical values (Figures 5C and D). Representative MS/MS spectra (Figures S9 and S10) demonstrate the simultaneous quantification of distinct peptides from two proteomes with different ratios in a single MS/MS scan.

Across technical triplicates, the median CVs for the yeast 5:1 and 10:1 quantification channels at the protein level were 3.09% and 2.08%, respectively. The probability density distributions for both channels are shown in Figure S11, indicating excellent reproducibility of the TMTproC-DIA method in complex proteomic contexts.

Additionally, we benchmarked TMTproC-DIA against the conventional TMTproC-DDA approach using the same dual-proteome sample. The TMTproC-DDA acquisition parameters are detailed in the Supplementary Methods. Across three technical replicates, the DIA approach yielded higher numbers of identified and quantified proteins and demonstrated improved reproducibility compared to the DDA method (Figure S12), highlighting the superior reproducibility and coverage of TMTproC-DIA.

Notably, slight ratio compression was observed at an MS/MS resolution of 60K (at *m*/*z* = 200), with the measured 1:1 channel deviating to 1.11. This distortion likely arose from unresolved overlaps among complementary ions from different peptides.^30,48^ For example, Figure S10A shows that the *m*/*z* difference between the 131N complementary ion of VTTHPLAK and the third isotopic peak of the 135N complementary ion of QNDITDGK is only 0.1 Da. While these closely spaced peaks were adequately resolved at 60K resolution (*m*/*z* 200), the minimum resolvable mass difference at *m*/*z* ∼ 1300 is approximately 0.055 Da at this resolution.^49^ Complementary ions with smaller mass differences may remain unresolved, leading to quantification inaccuracies.

To test this hypothesis, we analyzed the dual-proteome sample at a higher resolution of 120 K (at *m*/*z* 200). The median quantification ratios for yeast labeled at 1:5:10 improved to 0.98:4.92:10.01 at the protein level (Figure S13), indicating reduced ratio compression compared to results obtained at 60K resolution. However, higher-resolution scans increased cycle times, resulting in fewer identified and quantified proteins (Figure S13C). These findings suggest that a resolution of 60K (at *m*/*z* 200) offers a practical balance between quantification accuracy and proteome coverage.

## Discussions

This study demonstrates that TMTproC-DIA provides a practical approach to multiplexed DIA quantification with high precision and minimal interference. Unlike mass-defect or ultrahigh-resolution strategies that require long transients, TMTproC-DIA enables accurate, ratio-based quantification at moderate resolving power (60K at *m*/*z* 200). This level of performance is readily achievable on Orbitrap instruments and extends to ToF and Astral analyzers, which offer sufficient resolving power at high *m*/*z*.^50^ Importantly, TMTproC-DIA does not increase MS1 spectral complexity or multiplex unlabeled b/y ions in MS/MS, thereby preserving identification quality while conveying quantitative information through high-*m*/*z* complementary ions.

A key practical advantage of our design is that, for any given peptide, all three quantitative channels share the same MS1 precursor and are therefore isolated within the same DIA window during each duty cycle. This acquisition geometry is not attainable in nonisobaric multiplexing schemes such as plexDIA or BoxCarmax-DIA, where channels (or mass-difference offsets) may fall into different windows and scan cycles.^31,33^ By avoiding cross-window asynchrony, our strategy reduces channel-to-channel variance, which likely contributes to the observed low CVs. Additionally, because isobaric labeling inherently minimizes sample-handling heterogeneity and run-to-run variation, quantification accuracy is further improved compared to label-free quantification approaches.^11,12^

With 60 DIA windows and MS/MS at 60k (at *m*/*z* 200), the average duty cycle in our experiment was approximately 8.6 s. Under nanoflow LC conditions, where peptide baseline peak widths typically range from 30 to 60 s, this corresponds to 3–7 data points per peak. This sampling density supports robust ratio-based quantification and compares favorably with DDA-style approaches, which are constrained by dynamic-exclusion stochasticity. Notably, the duty cycle remains tunable and can be optimized to increase sampling density by adopting variable DIA windows and/or employing faster analyzers (e.g., ToF or Astral),^50,51^ which is expected to enhance proteome depth and improve precision.

Future directions may focus on improving identification while preserving complementary-ion-based quantification. First, integrating retention-time prediction models trained on TMT-labeled peptides into DIA scoring could enhance identifications.^52^ Second, selectively including unlabeled (“naked”) b/y fragments as sequence evidence may help increase identification rates in DIA data analysis. In parallel, the workflow remains compatible with advanced DIA configurations, including diaPASEF,^53^ narrow-window DIA^54^ and FAIMS-DIA,^55^ enabling practical adaptations that leverage each mode’s strengths to improve identification depth while maintaining multiplexing capability.

Finally, two limitations should also be noted. The cost of TMTpro reagents remains substantial and may constrain very large studies. In addition, throughput in the current triplex configuration is lower than in conventional high-plex TMTpro reporter-ion workflows. To explore plex scaling, we simulated different interchannel spacings (Figure S14). A 4 Da spacing minimizes residual overlap with simple [M+4] deconvolution. A 3 Da spacing is also feasible; however, given the current TMTpro isotopologue set (eight heavy isotopes in the mass-balancing group), a 3-Da layout does not increase throughput beyond triplex.^19,56^ A 2 Da spacing could potentially raise throughput to 5-plex but substantially increases isotopic overlap and requires more comprehensive deconvolution as well as careful evaluation of precursor-isotope isolation (full vs partial cluster) during deconvolution.^39^ Looking ahead, new tag chemistries with expanded isotope placement may enable higher plexing at larger effective spacings, reducing isotopic convolution while maintaining quantification precision.

## Conclusions

In this study, we introduced a novel 3-plex TMTproC-DIA strategy that effectively combines the high-throughput capability of isobaric labeling with the interference resistance of complementary ions. By designing complementary ions with a 4 Da spacing, we minimized isotopic peak overlap, thereby simplifying quantification and reducing errors associated with spectral complexity. Our method demonstrated high quantitative accuracy and precision across a dynamic range spanning an order of magnitude, as validated using tryptic BSA peptides and a human-yeast dual-proteome model. In summary, TMTproC-DIA offers a robust, versatile, and interference-resistant solution for high-throughput quantitative proteomics. Its compatibility with moderate instrument resolving powers and simplified data analysis makes it well-suited for a variety of biological and clinical applications, potentially paving the way for more comprehensive and scalable high-throughput DIA-based proteome analyses.

## Supplementary Material



## Notes

Method for the calculation of theoretical *m*/*z* values for complementary ions; detailed TMTproC-DDA MS instrumental method for performance comparison; method for TMTproC tag purity measurement; measured isotopic impurity ratios in TMTpro tags (Table S1); Workflow for TMTpro complementary-ion DIA data acquisition and processing (Figure S1); theoretical isotopic envelope distributions for TMTpro-labeled peptides with different complementary ion spacing (Figure S2); validation of complementary ion identification based on isotopic peaks (Figure S3); optimization of HCD fragmentation energy for complementary ion generation (Figure S4); distribution of the number of quantifiable peptides per protein across HCD collision energies for TMTpro labeled HeLa cell digest (Figure S5); representative MS/MS spectra of TMTpro-labeled BSA peptides at different labeling ratios (Figure S6 and Figure S7); distribution of quantified PSM counts by *m*/*z* values (Figure S8); representative MS/MS spectrum demonstrating simultaneous quantification of multiple peptides from combined human-yeast proteome within a single MS/MS scan. (Figure S9 and Figure S10); probability density distributions of CV values for yeast 5:1 and 10:1 quantification channels (Figure S11); comparison of reproducibility between TMTproC-DIA and TMTproC-DDA (Figure S12); influence of MS/MS resolution on quantification accuracy and proteome coverage (Figure S13); Theoretical isotopic envelope distributions for the TMTpro-labeled peptide LGEYGFQNALIVR under complementary-ion spacings of 2, 3, and 4 Da, across different mixing ratios of three channels (Figure S14). (PDF)
