# Supplementary material for: Multiplexed Data-Independent Acquisition-Based Proteomics Enabled by TMTpro Complementary Ions
Source: Anal Chem. 2025 Nov 26;97(48):26419–28. doi: 10.1021/acs.analchem.5c03563 (PMC12874209; doi:10.1021/acs.analchem.5c03563)
Supplement: Supplementary file 1 [file ac5c03563_si_001.pdf]

Supporting Information

Multiplexed Data-independent Acquisition-based Proteomics Enabled by TMTpro Complementary Ions

Zicong Wang[1], Peng-Kai Liu[2], Haiyan Lu[1] and Lingjun Li[1,2,3,4,5*]

[1] School of Pharmacy, University of Wisconsin-Madison, Madison, WI 53705, USA

[2] Biophysics Graduate Program, University of Wisconsin-Madison, Madison, WI, 53705, USA

[3] Department of Chemistry, University of Wisconsin-Madison, Madison, WI 53706, USA

[4] Lachman Institute for Pharmaceutical Development, School of Pharmacy, University of Wisconsin-Madison, Madison, WI, 53705, USA

[5] Wisconsin Center for NanoBioSystems, School of Pharmacy, University of Wisconsin-Madison, Madison, WI 53705, USA.

*Corresponding author: Lingjun Li

Email: lingjun.li@wisc.edu

Mailing address: 777 Highland Ave, Madison, WI 53705, United States

Tel: +1 608-265-8491, Fax: +1 608-262-5345

Table of Contents

Supplementary Methods

1. Calculation of Theoretical *m/z* of Complementary Ions

2. TMTproC-DDA MS Method for Performance Comparison

3.TMTproC Tag Purity Measurement and Correction

Supplementary Tables

Table S1. Isotopic impurity ratios for the commercially available TMTpro-tags

Supplementary Figures

Figure S1. Workflow for TMTpro complementary-ion DIA data acquisition and processing

Figure S2. Theoretical isotopic envelope distributions for a TMTpro-labeled peptide with 1Da and 4 Da complementary ion spacing at different labeling ratios.

Figure S3. Graphical description of complementary ions validation based on the presence of [M+1] isotopic peak.

Figure S4. Optimization of HCD fragmentation energy for complementary ion generation

Figure S5. Distribution of the number of quantifiable peptides per protein across different HCD collision energies for TMTpro labeled HeLa cell digest.

Figure S6. Representative MS/MS spectrum of TMTpro-labeled BSA peptide LGEYGFQNALIVR at different ratios

Figure S7. Representative MS/MS spectrum of TMTpro-labeled BSA charge 4+ peptide QEPERNECFLSHK at different ratios

Figure S8. Histogram illustrating the distribution of quantified PSM counts by their m/z values from the Yeast-HeLa dual-proteome sample

Figures S9 and S10. Representative MS/MS spectrum demonstrating simultaneous quantification of multiple peptides from human and yeast proteomes within a single MS/MS scan.

Figure S11. Probability density distribution of CV values for yeast quantification channels in TMTproC-DIA analysis of dual-proteome sample.

Figure S12. Comparison of the overlap of identified and quantified proteins across triplicates using TMTproC-DIA and TMTproC-DDA approaches.

Figure S13. Influence of MS/MS resolution on quantification accuracy and proteome coverage in TMTproC-DIA.

Figure S14. Theoretical isotopic-envelope distributions for a TMTpro-labeled peptide LGEYGFQNALIVR under complementary-ion spacings of 2, 3, and 4 Da across different mixing ratios of three channels.

Supplementary Methods

1. Calculation of theoretical m/z of complementary ions

The theoretical *m/z* values of complementary ions were calculated using the equations below, which consider the specific mass losses associated with TMTpro tags during complementary ion formation:

For TMTpro tag 135N:

$$m/z\_complementary = ((m/z\_precursor \times z) - 163.14648) / (z - 1)$$

For TMTpro tag 131N:

$$m/z\_complementary = ((m/z\_precursor \times z) - 159.13308) / (z - 1)$$

For TMTpro tag 126:

$$m/z\_complementary = ((m/z\_precursor \times z) - 155.12599) / (z - 1)$$

Here, z represents the charge state of the precursor ion. The constants 163.14648, 159.13308, and 155.12599 Da correspond to the specific mass losses (reporter ion plus neutral CO) associated with each TMTpro channel.

2. TMTproC-DDA MS Method for Performance Comparison

The TMTproC-DDA acquisition method was adapted from Johnson et al.[1] with slight modifications. Data were acquired in positive ion mode with an ionization voltage of 2,400 V and an ion transfer tube temperature of 275 °C. Full MS survey scans were acquired in the m/z range of 350–1400 using an Orbitrap detector at a resolution of 120,000 at m/z 200. The normalized automatic gain control (AGC) target was set to 40%, with a maximum injection time of 50 milliseconds.

Data-dependent acquisition was performed with a maximum cycle time of 3 seconds. Monoisotopic peak selection was enabled with Peptide Mode. Precursor ions with a charge state of 2–4 were selected. Dynamic exclusion was set for 60 seconds with a mass tolerance of ±10 ppm, while also excluding isotopes and different charge states of the isolated species.

MS/MS scans were acquired using a quadrupole isolation window of 0.4 Th and fragmented with higher-energy collisional dissociation (HCD) at a normalized collision energy (NCE) of 29%. Fragment ions were analyzed in the Orbitrap within the m/z range of 120–2655 at a resolution of 60,000 at m/z 200. The AGC target for MS/MS was set to 100%, with a maximum injection time of 120 milliseconds. The raw file was searched in MS Fragger and filtered at 1% FDR, then searched with the same python script with TMTproC-DIA but without the requirement of the presence of M+1 peak of complementary ion.

3. TMTproC Tag Purity Measurement and Correction

As described by Johnson et al.[1], the isotopic distribution of $^{13}C_6$-arginine was first measured using direct infusion-MS to establish a baseline for correction. $^{13}C_6$-arginine was then labeled with TMTpro reagents, ensuring complete reaction, and the isotopic distribution of the arginine-TMTpro complementary ions was analyzed using direct infusion-MS/MS. The -1 and +1 impurity ratios of TMTpro tag were calculated by solving linear equations based on the observed isotopic distribution before and after TMTpro labeling:

$$I_{(M)\_post} = k\times(y \times I_{(M)\_pre} + x \times I_{(M+1)\_pre} + z \times I_{(M-1)\_pre})$$
$$I_{(M+1)\_post} = k\times(z \times I_{(M)\_pre} + y \times I_{(M+1)\_pre})$$
$$I_{(M-1)\_post} = k\times(x \times I_{(M)\_pre} + y \times I_{(M-1)\_pre})$$
$$x + y + z = 1$$

Where $I_{(M)\_pre}$, $I_{(M+1)\_pre}$, and $I_{(M-1)\_pre}$ represent the measured intensities of the M, M+1, and M-1 monoisotopic peaks of $^{13}C6$-arginine prior to TMTpro labeling. $I_{(M)\_post}$, $I_{(M+1)\_post}$, and $I_{(M-1)\_post}$ represent the intensities of the measured complementary ion M, M+1, and M-1 monoisotopic peaks post TMTpro labeling. k is a coefficient accounting for signal intensity changes. x, y, and z are the purity correction factors for the TMTpro tags corresponding to the M-1, M, and M+1, respectively.

The calculated purity factors (x, y, z) are shown in Table S1. Using these calculated impurity ratios, a correction matrix was constructed and applied to the observed intensities of complementary ions to correct for isotopic impurities.

Supplementary Tables

Table S1. Isotopic impurity ratios for the commercially available TMTpro-tags in balancer region of complementary ion

TMTpro Channel	126	131N	135N
**M+1**	0.00%	2.96%	6.60%
**M**	97.23%	95.92%	93.40%
**M-1**	2.77%	1.12%	0.00%

Supplementary Figures

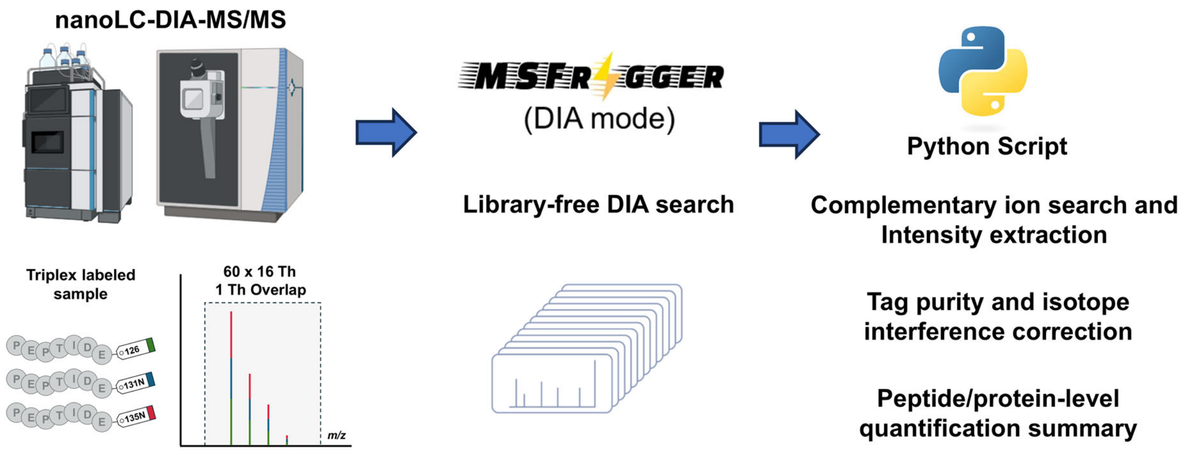

Figure S1. Workflow for TMTpro complementary-ion DIA data acquisition and processing

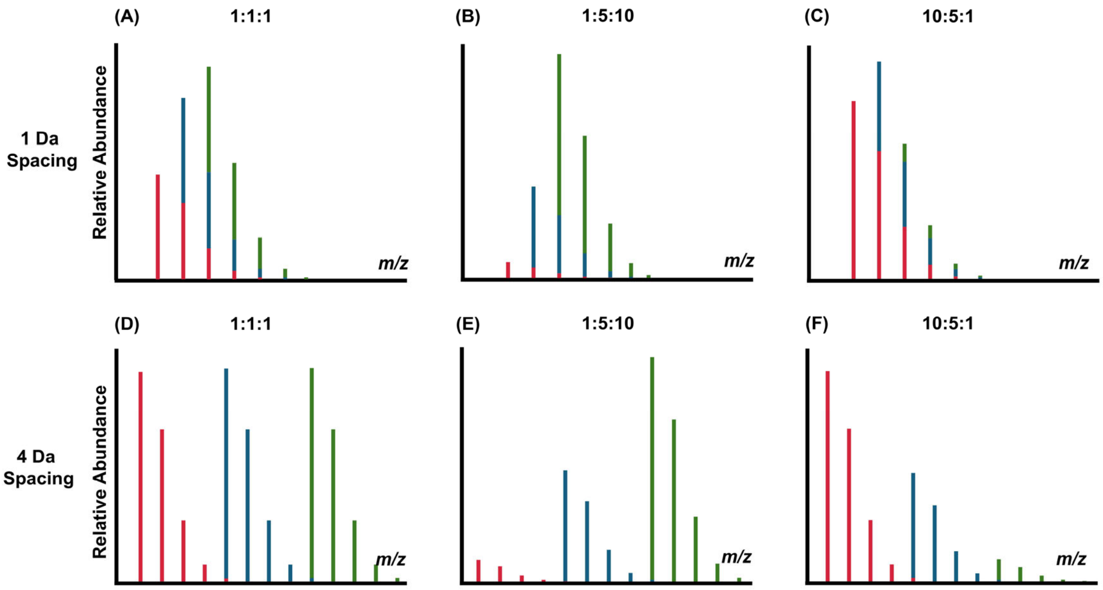

Figure S2. Theoretical isotopic envelope distributions for a TMTpro-labeled peptide (LGEYGFQNALIVR, $C_{68}H_{106}N_{18}O_{19}$) with 1Da and 4 Da complementary ion spacing at different labeling ratios. (A–C) Complementary ions with 1 Da spacing at labeling ratios of 1:1:1 (A), 1:5:10 (B), and 10:5:1 (C). Significant isotopic envelope overlaps are observed, especially for unbalanced ratios, necessitating complex deconvolution for accurate quantification. (D–F) Complementary ions with 4 Da spacing at labeling ratios of 1:1:1 (D), 1:5:10 (E), and 10:5:1 (F). The enlarged spacing effectively separates isotopic peaks, minimizing overlap and simplifying quantification.

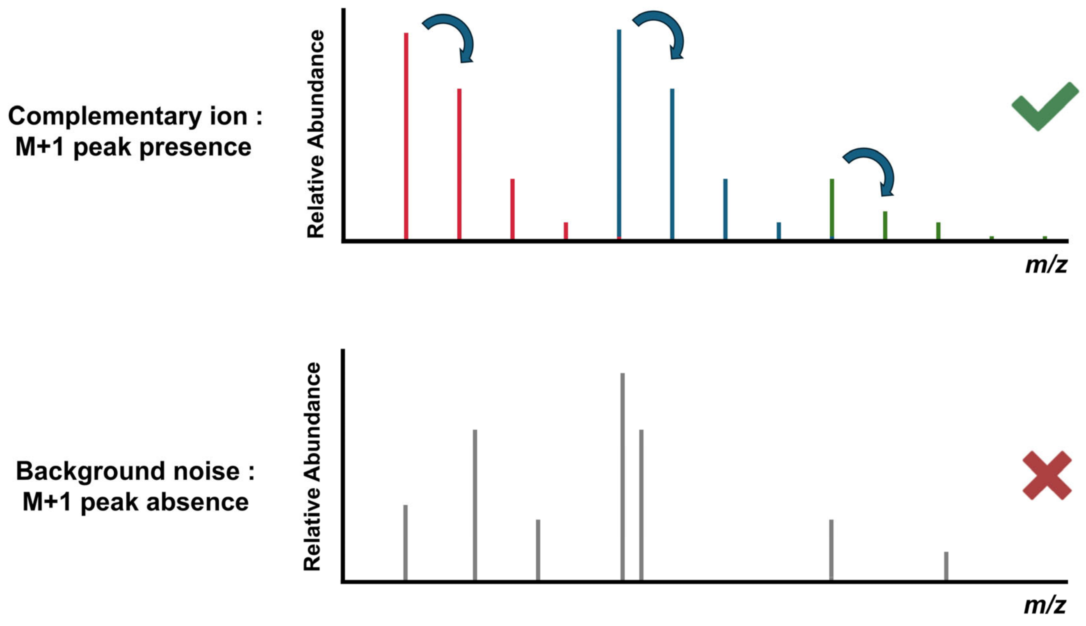

Figure S3. Graphical description of complementary ions validation based on the presence of [M+1] isotopic peak. Top panel**:** True complementary ions from the three distinct TMTpro channels (red, blue, and green) exhibit a consistent isotopic pattern, with both the [M+0] and [M+1] peaks present. This characteristic ensures their accurate identification and inclusion in quantitative analysis. Bottom panel: Background noise signals lack the [M+1] isotopic peak. These signals are excluded during analysis to avoid misidentification.

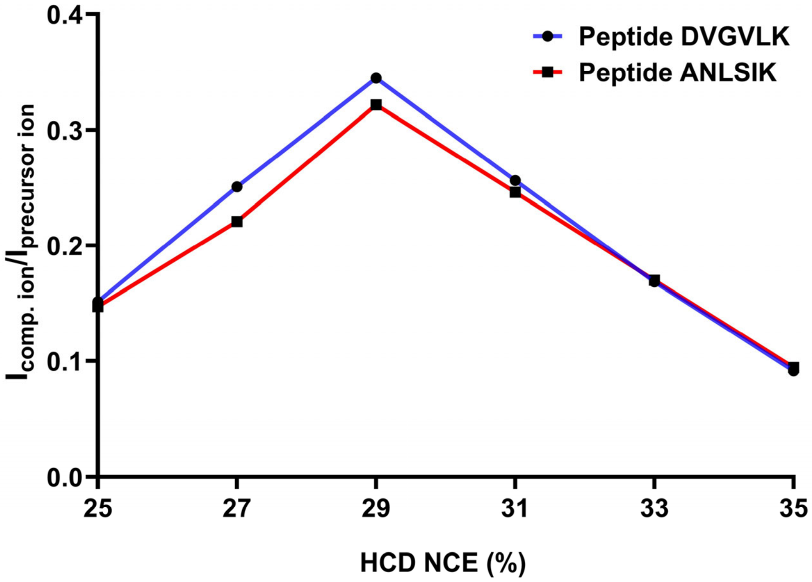

Figure S4. Optimization of HCD fragmentation energy for complementary ion generation. The ratio of complementary ion intensity to precursor ion intensity ($I_{comp.\ ion}/I_{precursor\ ion}$) was measured across a range of normalized collision energy (NCE) settings from 25% to 35% using peptide standards DVGVLK (blue) and ANLSIK (red). Both peptides exhibited peak intensity ratio at an NCE of 29%.

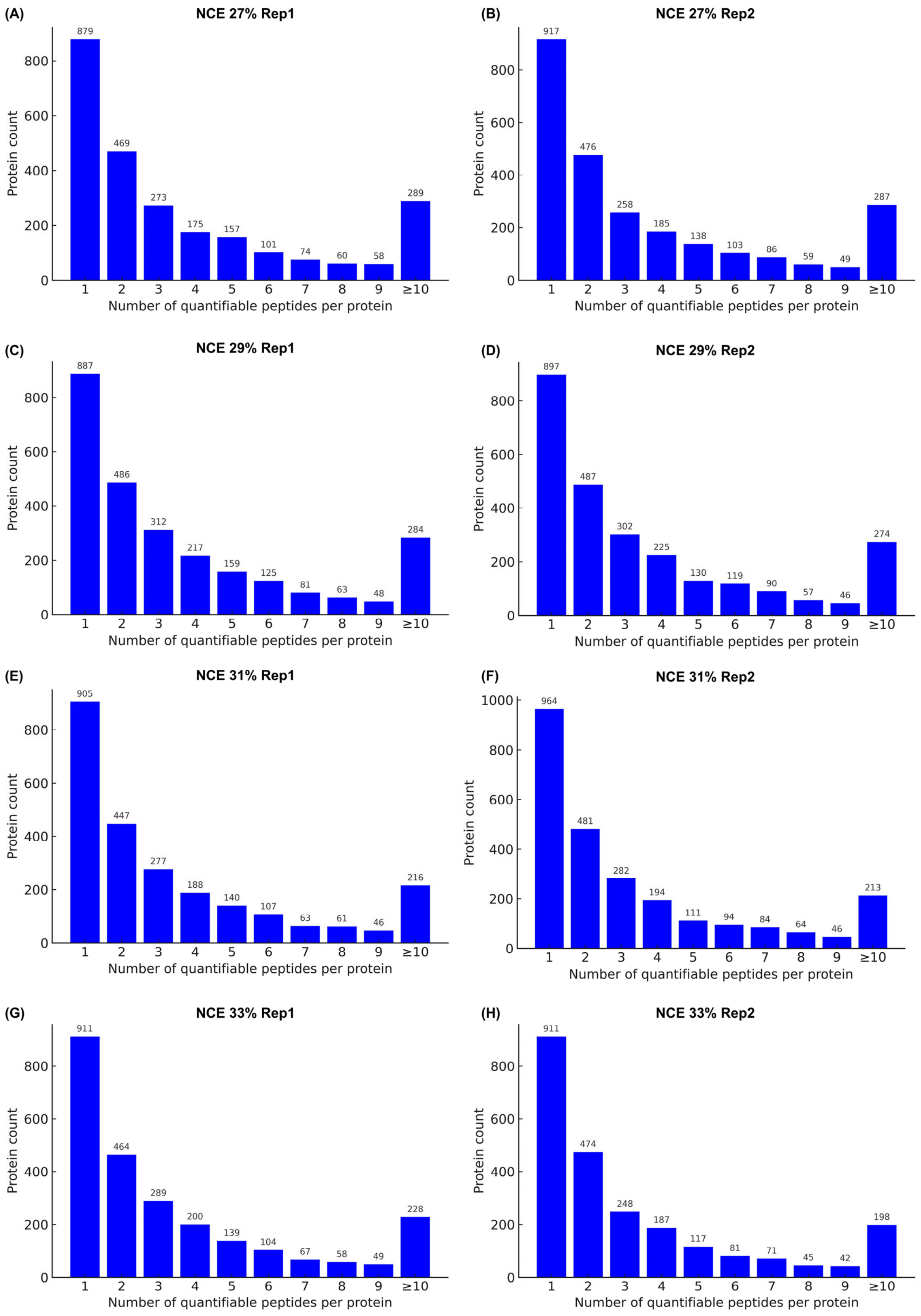

Figure S5. Distribution of the number of quantifiable peptides per protein across different HCD collision energies for TMTpro labeled HeLa cell digest.

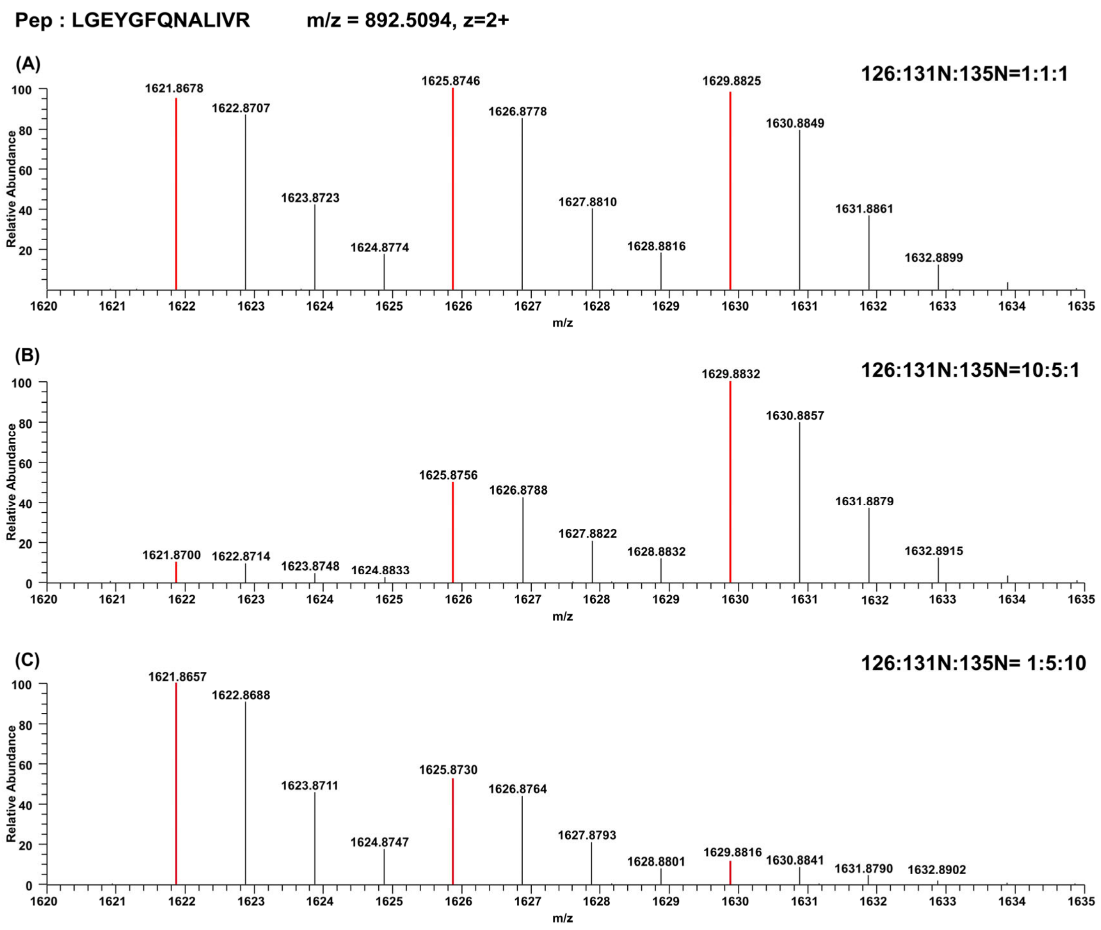

Figure S6. Representative MS/MS spectra of peptide LGEYGFQNALIVR (m/z = 892.5094, z = 2+) labeled with TMTpro channels 126:131N:135N at ratios of 1:1:1 (A), 10:5:1 (B), and 1:5:10 (C). The red lines highlight the M+0 peaks of complementary ion clusters. The measured complementary ion intensities closely match theoretical ratios across a wide range of labeling ratios.

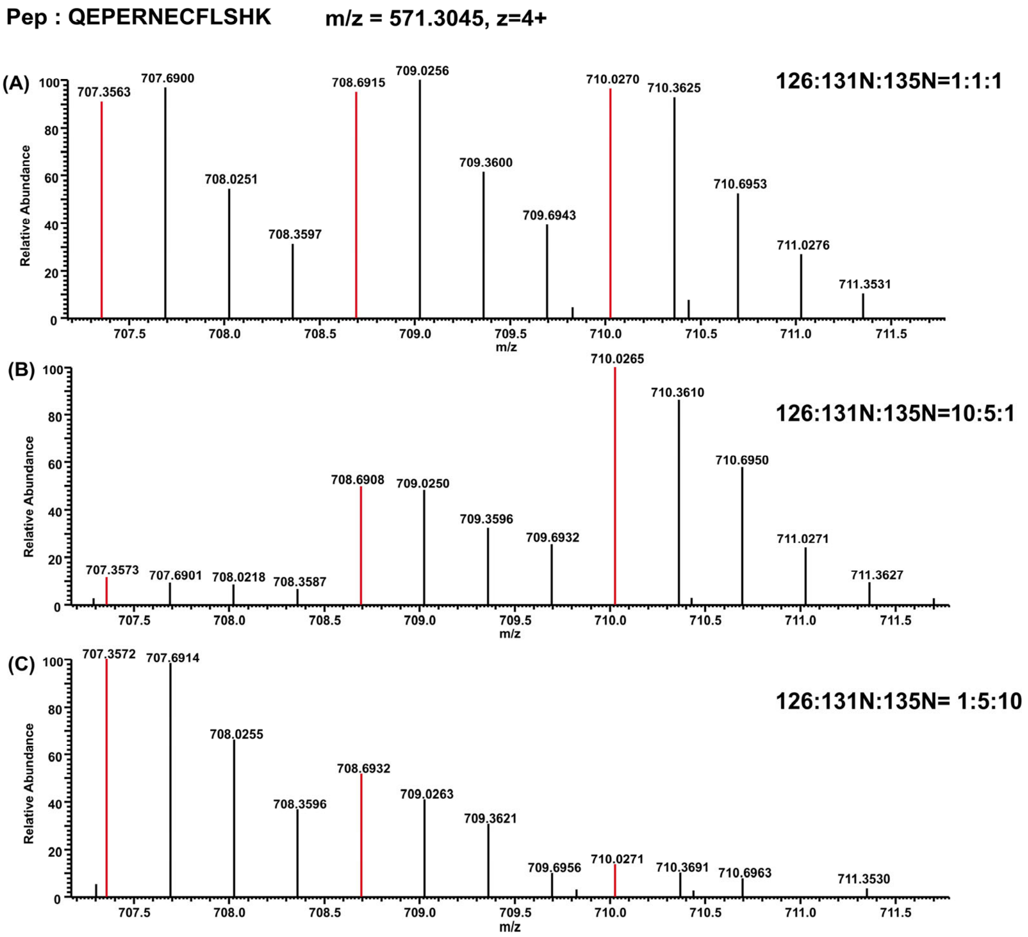

Figure S7. Representative MS/MS spectra of peptide QEPERNECFLSHK (m/z = 571.3045, z = 4+) labeled with TMTpro channels 126:131N:135N at ratios of 1:1:1 (A), 10:5:1 (B), and 1:5:10 (C). The red lines highlight the [M+0] peaks within the complementary ion clusters, showcasing accurate quantification across different labeling ratios.

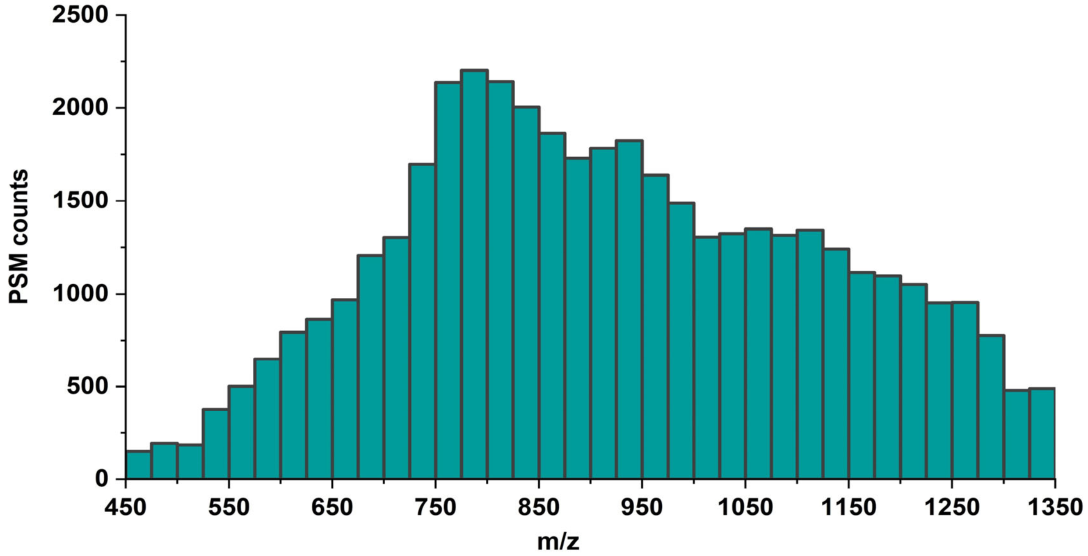

Figure S8. Histogram illustrating the distribution of quantified PSM counts by their m/z values from the Yeast-HeLa dual-proteome sample.

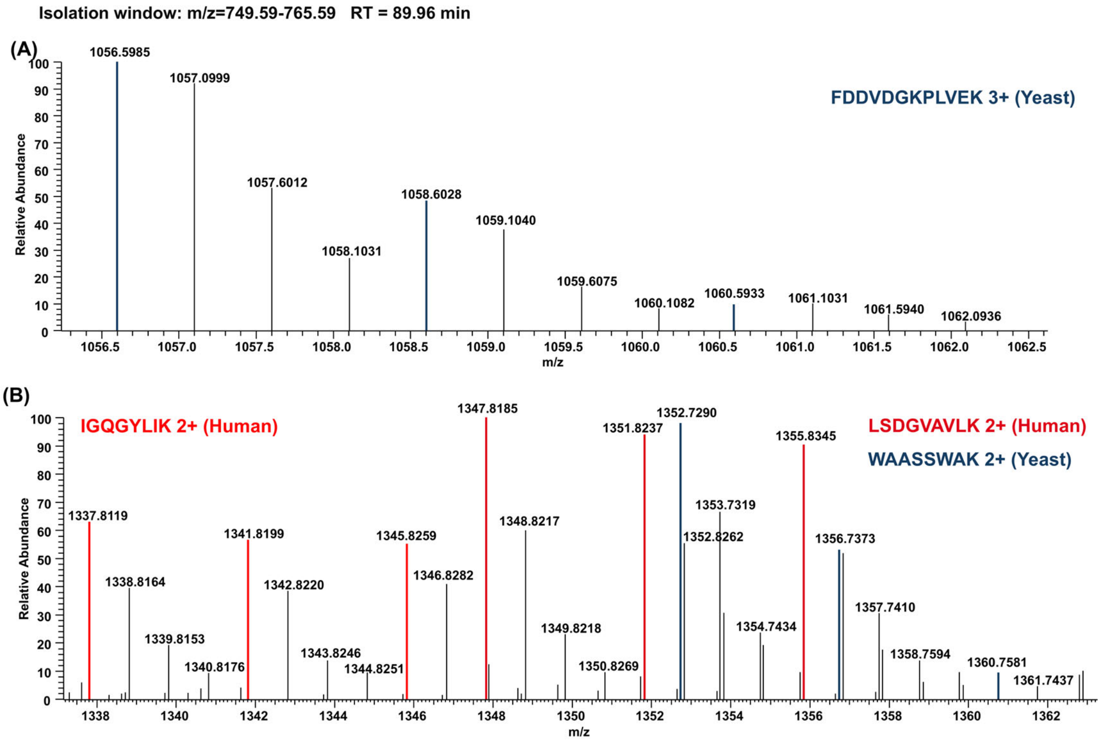

Figure S9. Representative MS/MS spectrum demonstrating simultaneous quantification of multiple peptides from combined human-yeast proteome within a single MS/MS scan. Panels (A) and (B) showed two separate m/z ranges from the same MS/MS spectrum, illustrating the complementary ion of peptides FDVDGKPLVEK (3+, yeast), IGQGYLIK (2+, human), LSDGVAVLK (2+, human), and WAASSWAK (2+, yeast). Complementary ion [M+0] peaks corresponding to each peptide are highlighted, with red and blue color indicating human and yeast peptides, respectively. These spectra showcase the TMTproC-DIA strategy's ability to simultaneously identify and quantify peptides from distinct proteomes within a single MS/MS scan.

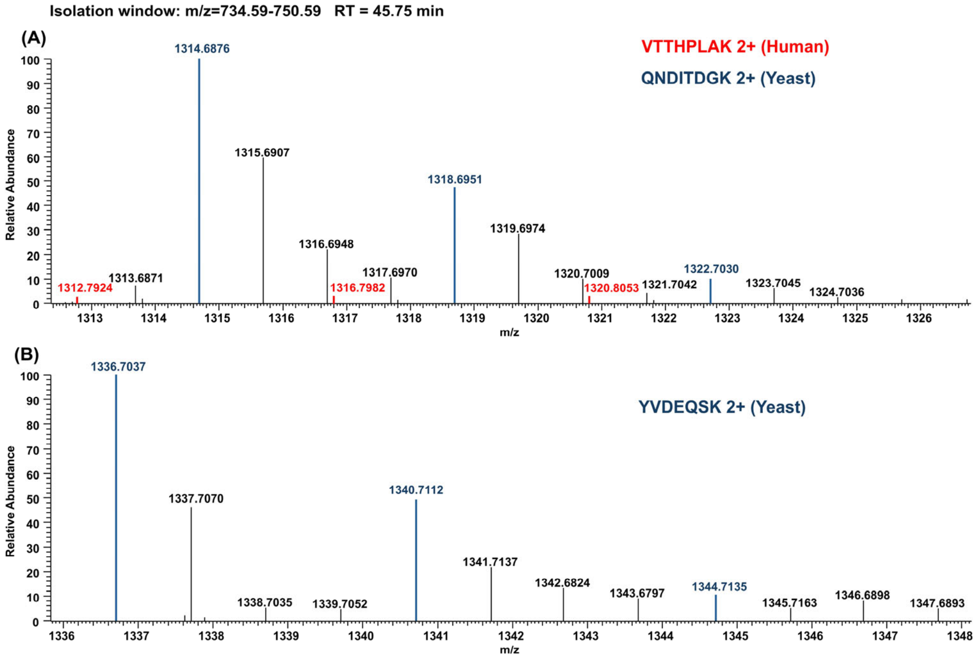

Figure S10. Representative MS/MS spectrum demonstrating simultaneous quantification of multiple peptides from combined human-yeast proteome within a single MS/MS scan. (A, B) showed two m/z ranges from the same MS/MS spectrum, illustrating the quantification of peptides VTTHPLAK (human, labeled at 1:1:1 ratio), QNDITDGK (yeast, labeled at 1:5:10 ratio), and YVDEQSK (yeast, labeled at 1:5:10 ratio) through complementary ions. Complementary ion [M+0] peaks corresponding to each peptide are highlighted, with red and blue color indicating human and yeast peptides, respectively.

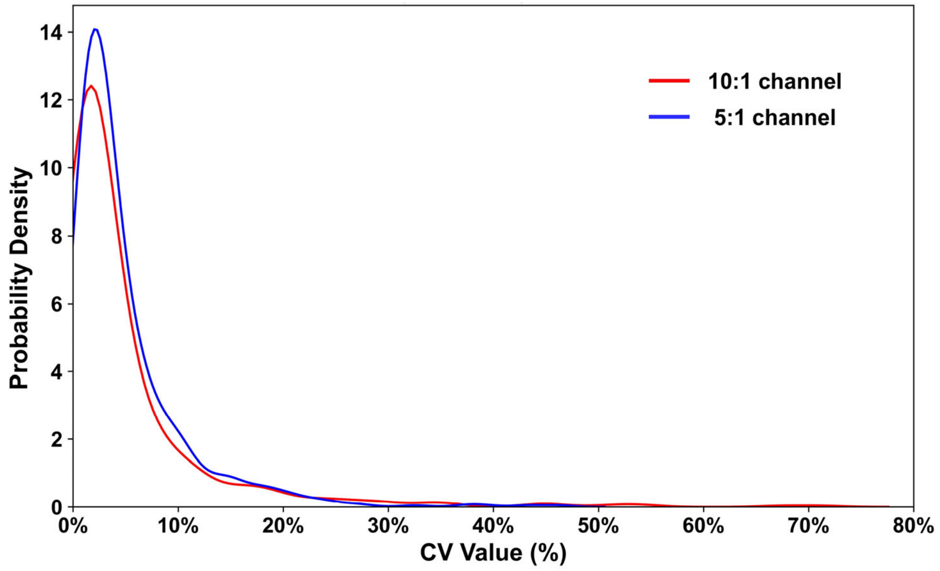

Figure S11. Probability density distribution of CV values for yeast quantification channels in TMTproC-DIA analysis of dual-proteome sample. The figure shows the probability density distribution of CV values for the 5:1 (blue line) and 10:1 (red line) quantification channels at the protein level across triplicates. Median CVs of 3.09% (5:1) and 2.08% (10:1) demonstrate high precision and reproducibility.

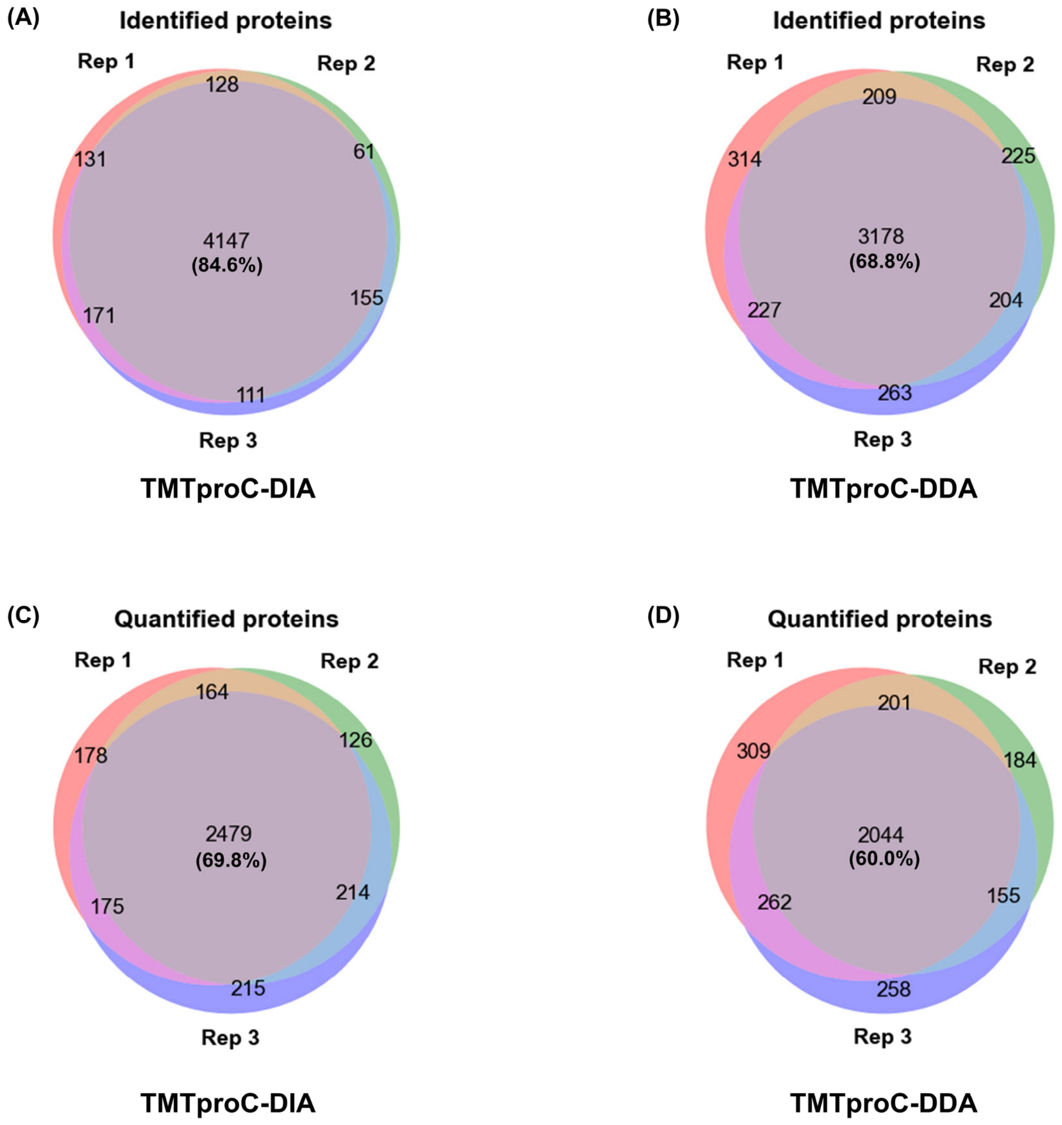

Figure S12. Comparison of the overlap of identified and quantified proteins across triplicates using TMTproC-DIA and TMTproC-DDA approaches. (A, B) Venn diagrams showing the overlap of identified proteins across three technical replicates for TMTproC-DIA (A) and TMTproC-DDA (B). (C, D) Venn diagrams showing the overlap of quantified proteins across three technical replicates for TMTproC-DIA (C) and TMTproC-DDA (D). TMTproC-DIA shows improved reproducibility and higher numbers of identified and quantified proteins compared to TMTproC-DDA.

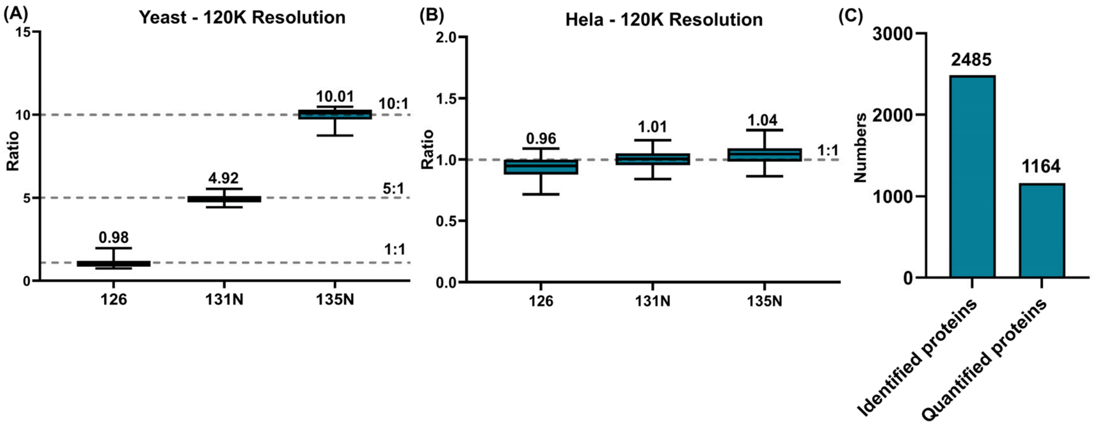

Figure S13. Influence of MS/MS resolution on quantification accuracy and proteome coverage in TMTproC-DIA analysis of human-yeast two-proteome sample. (A, B) Boxplots illustrating the quantification ratios of yeast proteins (A) and HeLa proteins (B) at 120K MS/MS resolution of the dual-proteome sample. Box plots demarcate the median (line), the 25th and 75th percentile (box), and the 5th and 95th percentile (whiskers). (C) Bar chart showing the total number of identified and quantified proteins at 120K MS/MS resolution.

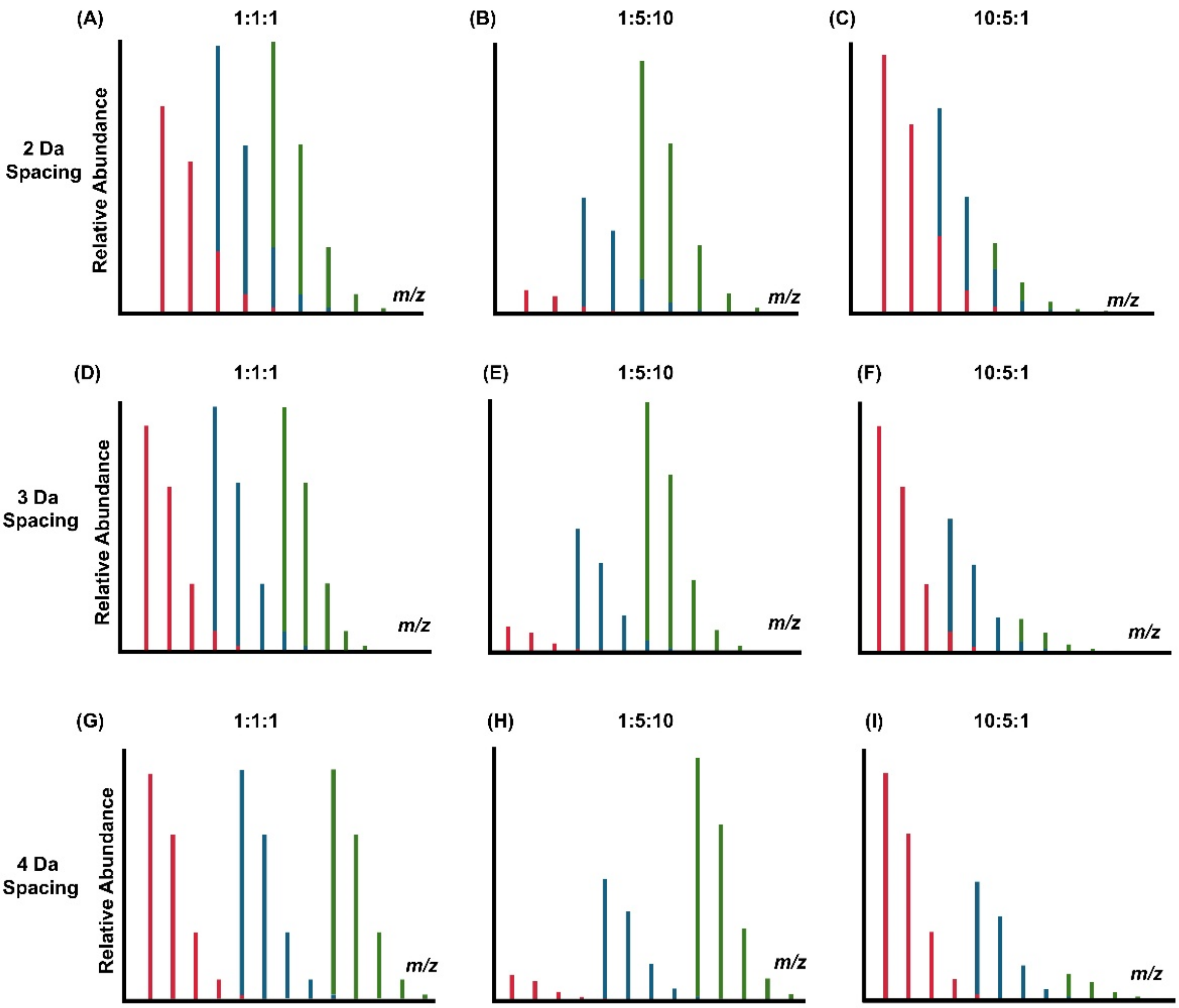

Figure S14. Theoretical isotopic-envelope distributions for a TMTpro-labeled peptide, LGEYGFQNALIVR ($C_{68}H_{106}N_{18}O_{19}$), under complementary-ion spacings of 2, 3, and 4 Da across different channel mixing ratios. Panels: A–C: 2 Da spacing; D–F: 3 Da spacing; G–I: 4 Da spacing. Ratios are 1:1:1, 1:5:10, 10:5:1 from left to right. Sticks depict theoretical isotope envelopes; red, blue, and green correspond to the three channels.

References

(1) Johnson, A.; Stadlmeier, M.; Wühr, M. TMTpro Complementary Ion Quantification Increases Plexing and Sensitivity for Accurate Multiplexed Proteomics at the MS2 Level. *J. Proteome Res.* **2021**, *20* (6), 3043–3052. https://doi.org/10.1021/acs.jproteome.0c00813.
